# Study Effects of Drug Treatment and Physiological Physical Stimulation on Surfactant Protein Expression of Lung Epithelial Cells Using a Biomimetic Microfluidic Cell Culture Device

**DOI:** 10.3390/mi10060400

**Published:** 2019-06-16

**Authors:** Ting-Ru Lin, Sih-Ling Yeh, Chien-Chung Peng, Wei-Hao Liao, Yi-Chung Tung

**Affiliations:** 1 Research Center for Applied Sciences, Academia Sinica, Taipei 11529, Taiwan; b98504030@ntu.edu.tw (T.-R.L.); sihlingyeh@gmail.com (S.-L.Y.); vp@gate.sinica.edu.tw (C.-C.P.); jamliao@gate.sinica.edu.tw (W.-H.L.); 2 College of Engineering, Chang Gung University, Taoyuan 33302, Taiwan

**Keywords:** microfluidic device, cell culture, organ-on-chips, lung epithelial cell, surfactant protein

## Abstract

This paper reports a biomimetic microfluidic device capable of reconstituting physiological physical microenvironments in lungs during fetal development for cell culture. The device integrates controllability of both hydrostatic pressure and cyclic substrate deformation within a single chip to better mimic the in vivo microenvironments. For demonstration, the effects of drug treatment and physical stimulations on surfactant protein C (SPC) expression of lung epithelial cells (A549) are studied using the device. The experimental results confirm the device’s capability of mimicking in vivo microenvironments with multiple physical stimulations for cell culture applications. Furthermore, the results indicate the critical roles of physical stimulations in regulating cellular behaviors. With the demonstrated functionalities and performance, the device is expected to provide a powerful tool for further lung development studies that can be translated to clinical observation in a more straightforward manner. Consequently, the device is promising for construction of more in vitro physiological microenvironments integrating multiple physical stimulations to better study organ development and its functions.

## 1. Introduction

Physical microenvironments play important roles in regulating various biological activities. In order to systematically investigate the effects of the physical microenvironments, various in vitro cell models have been developed and analyzed [1,2,3,4,5]. In a human body, the lung is an important respiratory organ, and various physical forces are involved during its development and operation. The cells within the lung experience different types of physical forces such as cyclic substrate deformation, surface tension, and hydrostatic pressure. In addition, during lung development in fetal stage, the physical stimulations are key factors regulating cell differentiation for proper lung function development. For instance, the stretch of tissues play a determinant role during lung development. It has been shown that intermittent and repetitive stretching of lungs induced by upper airway relaxation and the diaphragmatic contractions is an important factor for normal lung development in utero [6,7,8]. In addition, the larynx regulates the efflux of fetal intrapulmonary fluid produced by pulmonary epithelium from the trachea to the amniotic space to form positive pressure within the developing lungs [6,7]. However, due to the complicated anatomical organization of lung tissues, it is challenging to study in detail the effects of physical stimulations on the cells in vivo. The extensive study on lung development in vitro corresponding to physical stimulations is desired to decipher the underlying mechanisms for biomedical researches.

Recently, due to advantages provided by microfluidics, various microfluidic cell culture devices capable of reconstituting physiological microenvironments in organs, named organ-on-chips, have been developed for in vitro studies. Among the developed devices, several of them focus on reconstruction of physiological physical microenvironments in lungs for in vitro cell culture applications. For example, several devices are designed to apply cyclic strain and hydrostatic pressure on the cells to investigate mechanotransduction during breathing movement. The architecture of the devices emphasized on influences of application of substrate stretching on pneumocyte cells to investigate mechanisms behind the lung function [5,9,10,11,12,13], and analyses of cellular phenotypes, including orientation, attachment, and proliferation, are also performed after the cell culture [5,9,12]. Although the existing devices successfully provide physiological physical microenvironments for in vitro cell culture, and the results confirm the importance of physical stimulation for cell development, the existing devices mainly focus on providing single-type physical stimulations. In contrast, in vivo, multiple physical stimulations are often intertwined in physiological microenvironments. As a result, it is desired to integrate multiple physical stimulations within physiological ranges into single devices to construct in vitro cell culture models.

In this paper, we develop a microfluidic cell device capable of generating two physical stimulations, including hydrostatic pressure and cyclic substrate deformation, mimicking microenvironments during lung development. The device incorporates hydrostatic pressure and cyclic substrate deformation as physical stimulations for cell culture. The physical stimulations, including a hydrostatic pressure value, and the amplitude and frequency of the deformation, are designed according to the physiological conditions reported in previous studies. For the hydrostatic pressure, the device is designed based on the measured results from Nicolini et al., showing that the intra-amniotic pressure found in pregnancies with normal amniotic fluidic volume is 1–14 mmHg [14]. In terms of cyclic substrate deformation, Boyce et al. reported that the human fetal breathing rate is 30–90 times per minute [15]. In addition, Huh et al. indicated that the level of applied strain ranged from 5% to 15% matches normal levels observed in alveoli within whole lung in vivo [13]. As a result, the developed device is designed to provide the cyclic strain applied on cells at frequency of 30 stretches per minute and maximum surface strain of 9% under approximately 3 mmHg hydrostatic pressure to mimic physiological conditions.

To demonstrate the functionality provided by the designed biomimetic microfluidic cell culture device, we study expression of surfactant protein C (SPC) on type II pneumocytes under the steroid treatments and physical stimulations in this paper. SPC is an essential pulmonary surfactant protein maintaining normal operation of lungs. People lacking SPC tend to develop progressive interstitial lung disease [16]. In the experiments, the generated hydrostatic pressure and cyclic substrate deformation are numerically simulated and experimentally characterized. In the cell experiments, the biocompatibility of the device is first confirmed, and the device is further exploited to study the cell viability under combinations of drug treatments and physical stimulations. Furthermore, cell culture under various combinations of drug treatment and physical stimulations are performed to evaluate theirs effects on SPC expression of the cells. The results successfully confirm the capability of generating multiple physical stimulations and demonstrate the essentialness of physical stimulations on cellular responses. The developed device is believed to be able to pave the way to understand the cell behaviors under various physiological physicochemical stimulations.

## 2. Materials and Methods

### 2.1. Device Design and Fabrication

**Figure 1:**
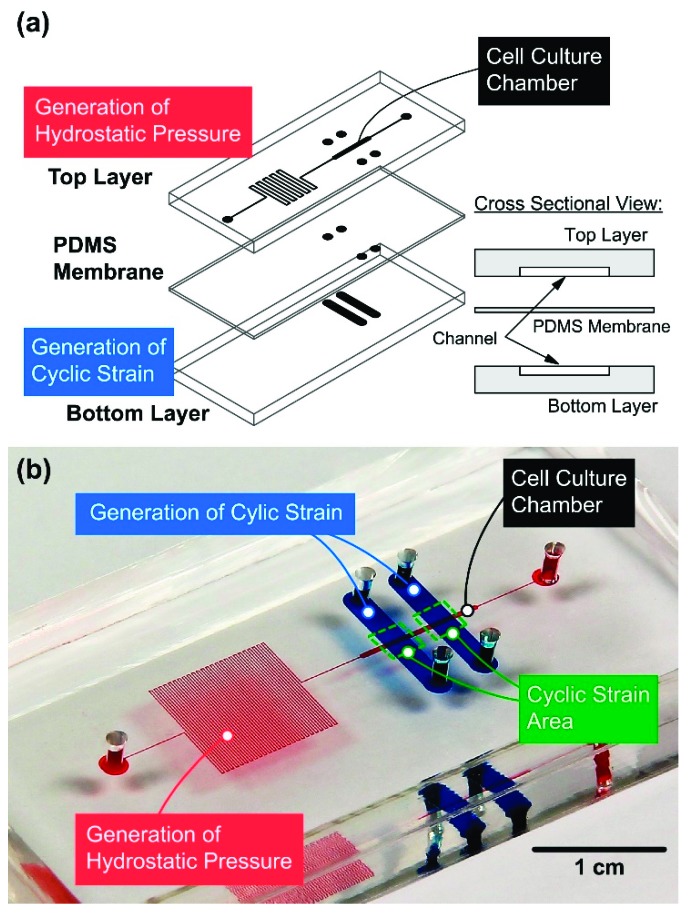
(**a**) Schematic of the designed microfluidic device to generate physical stimulations for lung epithelial cell surfactant protein expression studies. (**b**) Photo the fabricated device filled with colored food dyes (red: cell culture channel; blue: actuation channel.

The microfluidic device is designed to generate physical stimulations mimicking physiological conditions in a lung to study surfactant protein expression of lung epithelial cells. The microfluidic device designed in this paper is made of an elastomeric material polydimethylsiloxane (PDMS), due to its cell compatibility, optical transparency, and mechanical deformability [17]. The microfluidic device is constructed by two PDMS channel layers separated by a flexible PDMS membrane as illustrated in Figure 1a. The top layer is used for cell culture in a cellular microenvironment with well-defined physical stimulations, including hydrostatic pressure and cyclic surface strain. In order to generate hydrostatic pressure within a physiological range, growth medium is introduced into the top channel layer from an inlet, and flows through a cell culture chamber (width: 500 μm; height: 85 μm; length: 1 cm) followed by a serpentine-shape channel with relative high flow resistance towards an outlet. The bottom layer is exploited for actuation to deform the sandwiched membrane on which the cells are cultured. Deformation of the membrane is precisely controlled by infusing and withdrawing liquid in the actuation channel (width: 2.5 mm) with a designated volume and flow rate using a computer-controlled syringe pump [12].

The designed PDMS microfluidic device can be fabricated using soft lithography replica molding process [18,19]. For the fabrication of the two PDMS layers, the master molds fabricated on four-inch silicon wafers with 85 μm thick-negative photoresist (SU-8 2050, MicroChem Co., Newton, MA, USA) channel patterns are first prepared by photo lithography techniques. In order to prevent undesired adhesion between PDMS and the molds, their surfaces are silanized with 1H,1H,2H,2H-perfluorooctyltrichlorosilane (78560-45-9, Alfa Aesar, Ward Hill, MA, USA) in a desiccator at room temperature overnight. Degassed PDMS pre-polymer with base and curing agent (10:1 by weight) is then poured onto the molds and cured in a 60 °C oven overnight. The PDMS membrane with a thickness of 100 μm is prepared by spin coating the aforementioned PDMS pre-polymer on a four-inch silicon wafer at 100 rpm, and the membrane is then cured in the oven overnight.

To assemble the device, the inlet and outlet to the cell culture channel on the top layer are first made using a 2 mm-diameter biopsy punch (Miltex, York, PA, USA). The top layer is then irreversibly bonded to the membrane using oxygen plasma surface treatment at 90 W for 40 s (PX-250, Nordson MARCH Co., Concord, CA, USA). The inlets and outlets to the actuation channels on the bottom layer are then made by the biopsy punch through the assembled layer. The bottom layer is then bonded to the assembled top layer using the same surface treatment. Last, the bonded device is placed in the 60 °C oven overnight to promote adhesion and cell compatibility. A photo of the fabricated microfluidic device filled with colored food dyes is shown in Figure 1b.

### 2.2. Experimental Setup

**Figure 2:**
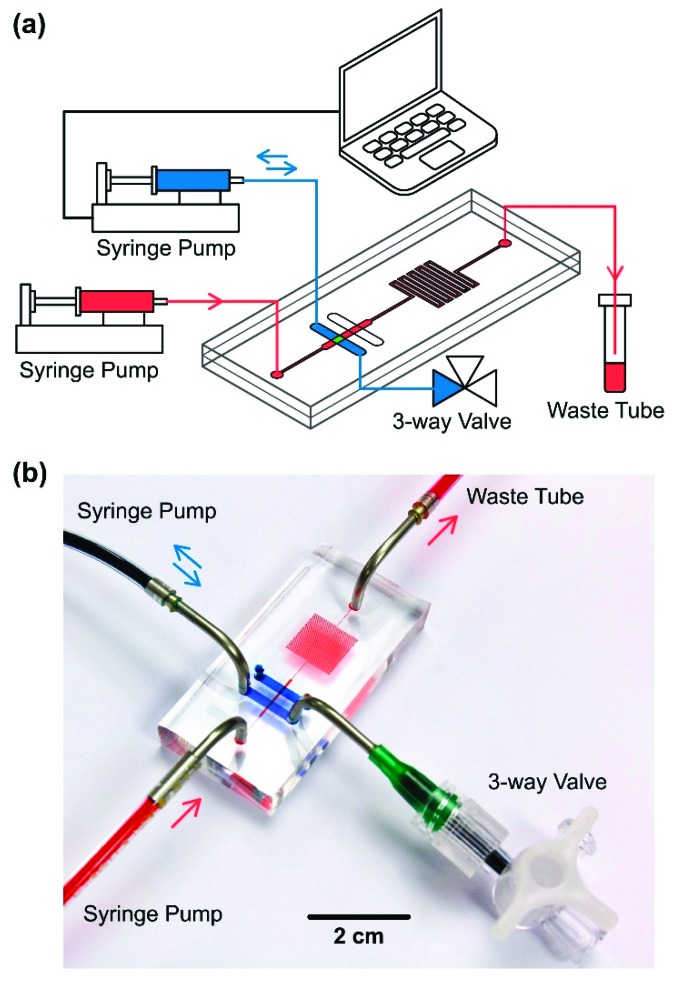
(**a**) Schematic and (**b**) photo of the experimental setup of the microfluidic cell culture device capable of mimicking physical stimulations within lungs.

To perform cell culture under well-controlled physical microenvironments using the developed microfluidic device, experimental setup as shown in Figure 2 is constructed. In the setup, a syringe pump (Fusion 400 classical Syringe Pump, Chemyx Inc., Stafford, TX, USA) is connected to the cell culture chamber inlet to continuously pump cell growth medium for perfusion of the cell culture. In order to generate cyclic substrate deformation, a computer-controlled syringe pump (Fusion 200 classical Syringe Pump, Chemyx Inc., Stafford, TX, USA) and a three-way valve are connected to the inlet and outlet of the actuation channel, respectively. To generate a precise cyclic surface strain for the cell culture, the syringe pump is controlled by a LabVIEW program (Ver. 2012, National Instruments, Co., Austin, TX, USA) to periodically infuse and withdraw deionized water with specific volume in and from the actuation channel. To assure the consistent membrane deformation during the experiments, the three-way valve connected to the outlet is exploited to eliminate the air bubbles trapped inside the actuation channel.

### 2.3. Device Simulation and Characterization

In order to confirm the agreement of the physical microenvironments established within the microfluidic device with the physiological ones, numerical simulation and experimental characterization are performed in this paper. First, a three-dimensional (3D) finite element analysis (FEA) model is constructed using a commercially available FEA simulation software (COMSOL Ver. 4.3b, COMSOL Inc., Burlington, MA, USA) to estimate the hydrostatic pressure inside the cell culture chamber in the top layer during the cell perfusion culture. In the model, tetrahedral elements (number of iterations: 4; maximum element depth to process: 4) with auto mesh, and the material properties of water are exploited for flow simulation. The channel geometries are set to be identical to the mask design. For boundary conditions, no-slip conditions are applied on all the channel walls, and the inlet and outlet are set to be a flow with a uniform flow rate of 0.1 µL/min and atmosphere pressure, respectively. In the FEA software, Navier–Stokes equations are utilized as governing equations to solve the constructed fluidic model.

In order to mimic the physical stimulations of fetal breathing movements of lungs, the cyclic substrate deformation frequency at 30 stretches per minute and maximum surface strain of 9% are tested in the experiments. The deformation and the surface strain are experimentally characterized and numerically simulated to estimate the strain generated on the membrane top surface. For the characterization, fluorescein sodium salt solution (F6377, Sigma- Aldrich Co., St Louis, MO, USA) with concentration of 50 µg/mL is introduced into both cell culture and actuation channels located in the top and bottom layers. The solution is infused into and withdrawn from the actuation channel with a flow rate of 1.5 mL/min for 1 s to deform the membrane, and the deformation is observed through cross-sectional imaging using confocal microscopy (TCS SP5, Leica Microsystems, Wetzlar, Germany).

To estimate surface strain on the membrane top surface with the specific actuation conditions, the FEA simulation based on the governing equation stating divergence of stress equals the volume force is also performed. A 3D model simulating the membrane (0.5 mm × 2 mm × 0.1 mm in width × length × thickness) with the auto-meshed tetrahedral elements and boundary conditions of fixed edges is constructed in the software. In the simulation, the Young’s modulus, Poisson’s ratio, and density of the PDMS membrane are set as 7.5 × 10^5^ Pa, 0.49, and 9.2 × 10^2^ kg/m^3^, respectively [5]. A series of uniformly distributed force is applied on the membrane bottom surface in the simulation to yield the same membrane maximum displacement observed in the experimental characterization.

### 2.4. Cell Culture and Seeding

In the cell experiments, carcinomic human alveolar basal epithelial cells (A549, ATCC, Manassas, VA, USA) are utilized to study pulmonary surfactant protein expression using the developed microfluidic cell culture device. The A549 cell line has been broadly used as a model of the type II pneumocytes for in vitro experiments [20]. The A549 cells are cultured using growth medium based on F-12K medium (Gibco 21127, Invitrogen Co., Carlsbad, CA, USA) with 10% *v/v* fetal bovine serum (FBS) (Gibco 10082, Invitrogen) and 1% *v/v* antibiotic–antimycotic (Gibco 15240, Invitrogen). The stocks are cultured in T25 cell culture flasks (Nunc 156367, Thermo Scientific Inc., Rochester, NY, USA) maintained at 37 °C in a humidified incubator under 5% CO_2_ in air, and the cells are passaged by dissociation using trypLE (Gibco 12604, Invitrogen). Before introducing the cells into the microfluidic devices, the devices are sterilized by UV irradiation and then treated with O_2_ plasma at 90 W for 40 s to make channel surfaces hydrophilic. The cell culture channels are then coated with extra-cellular matrix (ECM) protein, fibronectin (F2006, Sigma-Aldrich Co., St Louis, MO, USA), with concentration of 100 μg/mL in Dulbecco’s Phosphate-Buffered Saline (DPBS) (Gibco 14190, Invitrogen) overnight inside the incubator to promote cell adhesion. For microfluidic cell culture experiments, A549 cell suspension with volume of 100 μL and density of 2 × 10^7^ cells/mL in the growth medium is prepared. The cell suspension is then manually injected into the device using a 1 mL disposable syringe (Korea Vaccine Co. Ltd., Seoul, Korea) with a 14-gauge stainless steel needle (Jensen Global Inc., Santa Barbara, CA, USA). To assure the A549 cells well attach onto the PDMS membrane for the experiments, the cells are cultured in the device under static conditions in the cell incubator overnight before the drug treatment and physical stimulation experiments.

In order to compare effects of conventional drug treatments with physical stimulation on surfactant protein expression, a steroid drug, dexamethasone, is also tested in the experiments for comparison. Dexamethasone has been reported to be capable of stimulating fetal lung maturation and promoting surfactant secretion [21,22]. In the experiments, 1 μM dexamethasone (D4902, Sigma-Aldrich) in the growth medium is exploited to treat the cells in the microfluidic device to study the surfactant secretion resulted from the steroid [10]. In addition, to systematically investigate effects of the dexamethasone treatment, physical stimulation, and their combinations on the pulmonary surfactant protein expression, four sets of experiments are conducted in this study: (1) Device A: cell culture with neither supplement of dexamethasone nor physical stimulation as control; (2) Device B: cell culture with growth medium containing 1 μM dexamethasone; (3) Device C: cell culture with the physical stimulation; and (4) Device D: cell culture with 1 μM dexamethasone and the physical stimulation. The experiments are conducted inside a conventional cell incubator, and the growth medium is continuously perfused with flow rates of 0.1 μL/min during the experiments. All the experiments are repeated three times for statistical analysis. In addition, viabilities of the A549 cells cultured in the experiments under various conditions are characterized to investigate the cell compatibility of the device and effects of the treatments on cell viability. The viabilities are quantitatively evaluated using a fluorescence LIVE (green)/DEAD (red) Viability/Cytotoxicity Kit (L3224, Invitrogen) containing Calcein AM (2 μM) and ethidium homodimer-1 (2 μM). The fluorescence nuclei staining, bisbenzimide H33342 trihydrochloride (1 μg/mL) (B2261, Sigma-Aldrich) in DPBS, is also performed for cell quantification.

### 2.5. Analysis of Surfactant Protein Expression

Surfactant protein C (SPC) is one of the pulmonary surfactant proteins. SPC uniquely expressed in the alveolar type II cells, which are responsible for production and secretion of pulmonary surfactant [23]. In the cell experiments, immunocytochemical staining of SPC on the A549 cells is performed to study surfactant protein expression of the cells under drug treatment and physical stimulation. The immunocytochemical staining is conducted by first washing cells using DPBS and fixing the cells within the microfluidic devices by 4% paraformaldehyde (PFA) (158127, Sigma-Aldrich Co., St. Louis, MO, USA) for 20 min. The fixed cells are then washed with DPBS containing 0.1% *v/v* Tween 20 (#161-0781, Bio-Rad Laboratories, Inc., Hercules, CA, USA) and permeabilized using 0.1% Triton X-100 (T9284, Sigma-Aldrich) for 5 min. The cells are then blocked in 3% bovine serum albumin (BSA) (A7906, Sigma-Aldrich) in DPBS for 2 h at room temperature. The cells are then stained with anti-prosurfactant protein C antibody (ab40879, Abcam, Cambridge, UK) at a ratio of 1:250 in DPBS at 4 °C overnight. Last, donkey anti-rabbit IgG secondary antibody (A21206, Invitrogen), at a ratio of 1:1000, is used with an additional 1-h incubation at ambient temperature after washing the first antibody with PBST (0.1% (*v*/*v*) Tween 20 in D-PBS) for visualization. The nuclei stain with 2 μg/mL DAPI is also performed on the cells for the following analysis.

An inverted fluorescence microscope (AF7000, Leica Microsystems Ltd.) equipped with a charge-coupled device (CCD) camera (ORCA-R2, Hamamatsu Photonics, Shizuoka, Japan) is exploited to capture brightfield and fluorescence images of the cells. To quantify the protein expression level from the fluorescence images, a monochromatic channel of the green fluorescence images is process by a code written in MATLAB R2014a (MathWorks, Inc., Natick, MA, USA). In the code, average fluorescence intensity per pixel from all detected pixels within the cells, which are identified by pixels with intensities above a threshold, is calculated. The calculated average intensity of each experiment is then normalized to the average intensity of the control experiment (Device A) conducted at the same time to eliminate possible experimental bias from different experiments. For statistical analysis, three independent sets of experiments are conducted, and the analysis of variance (ANOVA), followed by Holm–Sidak tests, is exploited to compare the normalized quantitative fluorescence intensities corresponding to the four conditions.

## 3. Results and Discussion

### 3.1. Device Characterization

**Figure 3:**
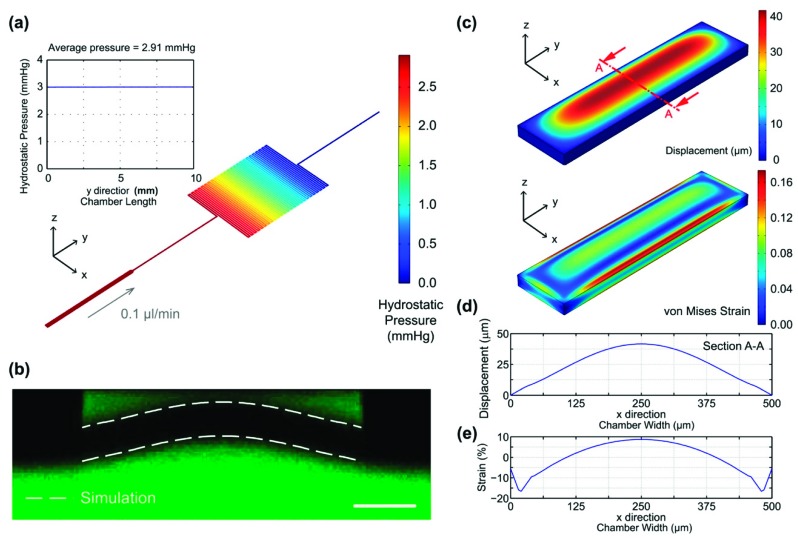
(**a**) The simulated hydrostatic pressure within the entire device. The inset demonstrates the uniform hydrostatic pressure along the flow direction in the cell culture chamber. (**b**) Confocal microscopic image showing the cross-section of the device with fluorescein solutions filled in the cell culture chamber and the actuation channel along the width of the cell culture chamber. The scale bar is 200 μm. The simulated deformations of the membrane are plotted in dotted lines showing the good agreement between the simulated and experimental results. (**c**) Contour plots of the displacement and von Mises strain distributions of the entire membrane across the cell culture chamber. (**d**) Plots of the displacement and strain along the top surface of the membrane across the width of the cell culture chamber.

The physical microenvironments established within the microfluidic device are characterized by numerical simulation and experimental measurements. First, the hydrostatic pressure within the cell culture chamber during the perfusion cell culture with flow rate of 0.1 μL/min is simulated using the aforementioned FEA model. Figure 3a shows the simulated hydrostatic pressure distributions within the entire top layer channel. The average hydrostatic pressure within the cell culture chamber along the flow direction in the cell culture chamber is 2.91 mmHg with variation less than 0.01 mmHg, which is within the measured physiological values from previous research [14]. In addition, the shear stress within the cell culture chamber was also estimated using the simulation. The result show that the average shear stress on the chamber walls on which the cells are cultured is approximately 1.28 × 10^−2^ dyn/cm^2^, which is negligible for the cells. The results suggest that well-controlled hydrostatic pressure with value mimicking physiological ones can be established for perfusion cell culture using the designed device.

In addition, deformation of the membrane within the microfluidic device is experimentally characterized using the confocal microscopy. Figure 3b shows the cross-sectional fluorescence confocal microscopic image of the deformed membrane when both top and bottom channels are filled with the fluorescein solution. To validate the numerical simulation results, the displacements of the top and bottom surfaces of the membranes are also plotted as white dotted lines and overlaid with the fluorescence image. The simulated deformation curves agree well with the experimental observation suggesting that the numerical simulation provides reasonably accurate prediction and can be used for the surface strain estimation. Figure 3c shows the simulated out of plane displacement and von Mises strain distribution of the entire membrane. The displacements of four membrane edges are zero due to the fixed boundary conditions, and the largest deformation greater than 40 μm occurs in the central part of the membrane. For the von Mises strain, the value decreases from a maximum value (16%) to zero and then increases from zero to 10% from the edge to the center along the channel width. Figure 3d shows the out of plane displacement (z direction) of the membrane at a cross-section located at the middle of the cell culture chamber along the flow direction (Section A-A). The average and maximum displacements are 24.7 μm and 41.6 μm, respectively. The results confirm that the membrane does not attach to the top surface of cell culture chamber resulting in cell damage because the channel height (85 μm) is larger than the sum of cell thickness and maximum z-displacement of the membrane.

To investigate the mechanical strain on the surface on which the cells are cultured, the normal strain in the direction along the width of the cell culture chamber (x-direction) is plotted as Figure 3e. From the edge to the center, the normal strain decreases slight to a minimum (−16%), and then gradually increases to a maximum (8.7%). Most of the simulated normal strains range from 2% to 8.7% across the width of the cell culture chamber (x = 125–375 μm), where the stretched cells are investigated. The designed strains ranges are within reported values from previous literatures. For instance, Liu et al. and Quinn et al. adopted cyclic biaxial 5–25% stretch to simulate the fetal breathing movement [9,13,24]. The simulated results confirmed that the device is capable of providing cultured cells physical stimulation of surface strain within the physiological ranges.

### 3.2. Cell Viability Characterization

**Figure 4:**
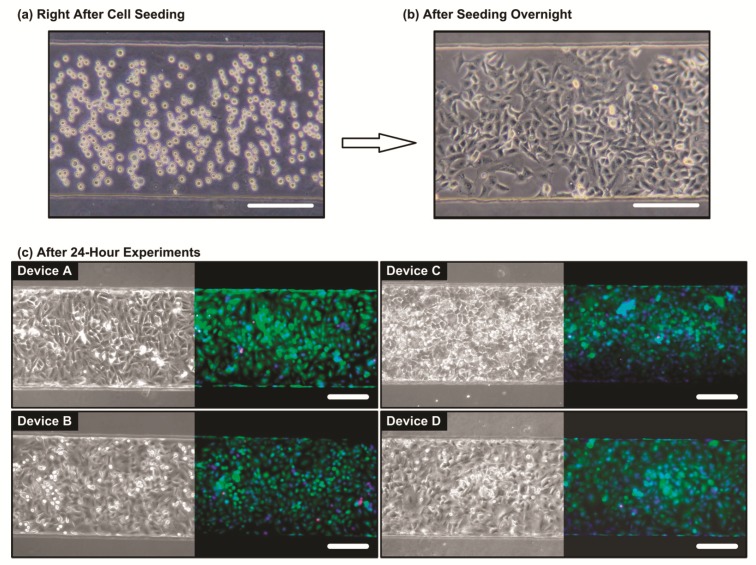
The microscopic images of the A549 cells (**a**) right after seeding into the microfluidic device, and (**b**) after culturing in the microfluidic device overnight. After the overnight culture, the A549 cells attach well onto the cell culture chamber substrate. (**c**) Bright field phase and live (green)/dead (red)/nuclei (blue) fluorescence images of the A549 cells inside the culture chamber after 24-h cell experiments under the 4 different conditions. Device A: control experiment in the growth medium; Device B: cell culture in the growth medium containing 1 μM dexamethasone; Device C: cell culture in the growth medium with the physical stimulations; and Device D: cell culture under the combination of dexamethasone treatment and physical stimulations. The scale bars are 200 μm.

To confirm the cell compatibility of the device and the cell experiments, the cells cultured inside the microfluidic device are observed using the microscope before and after the experiments. Figure 4a,b show phase contrast microscope images of the A549 cells right after seeding and after seeding overnight, respectively. The images demonstrate that the A549 cells can well adhere onto the fibronectin-coated PDMS membrane. To further estimate the cell viabilities after the experiments, the cells cultured in the device are stained with live(green)/dead(red) fluorescence dyes after 24-h culture under the combinations of the 1 μM dexamethasone and physical stimulations. As shown in Figure 4c, the fluorescence image indicates majority of the cells (>95%) show green fluorescence after the 24-h culture under the four combinations of 1 μM dexamethasone and physical stimulations within the device (Device A, B, C, and D), and there are no significant differences between the cell viabilities under different experimental conditions. The results suggest that the device possesses great biocompatibility, and the drug treatment and the physical stimulation do not significantly affect the cell viability.

### 3.3. Expression of SPC

**Figure 5:**
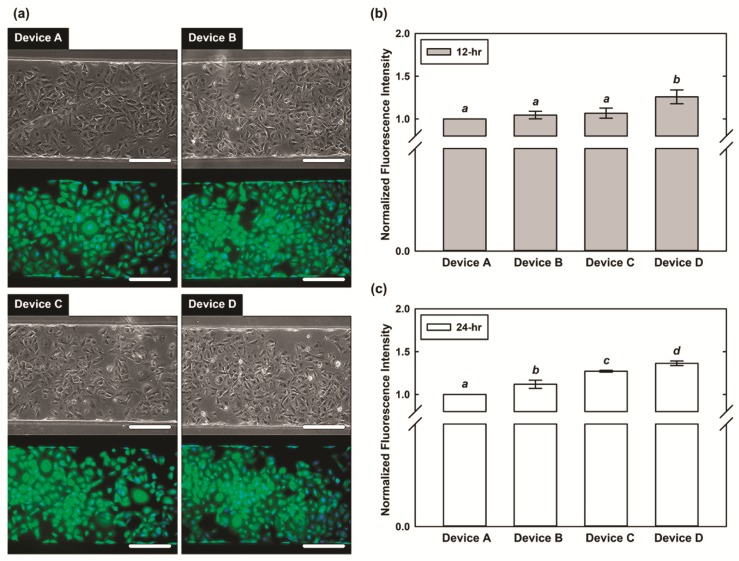
(**a**) Bright field phase and fluorescence images of the A549 cells cultured within the microfluidic devices with different combinations of steroid treatment and physical stimulation. Device A: neither steroid treatment nor physical stimulation (control); Device B: only steroid treatment; Device C: only physical stimulation; Device D: steroid treatment and physical stimulation. (**b**,**c**) Normalized average fluorescence intensity showing SPC expression of the A549 cells after 12- and 24-h experiments under the four different conditions, respectively. The fluorescence intensities showing SPC expression levels with statistically significantly different are designated with different letters (a, b, c, d = *p* < 0.05).

To investigate the SPC expression of the A549 cells, fluorescence staining with microscopic imaging is performed in the experiments. Figure 5a shows the brightfield phase contrast images and fluorescence images stained with pulmonary SPC under the four different conditions after the 12-h experiments. The A549 cells cultured in the microfluidic device for 12 h show similar morphologies under the four different conditions. The results suggest that neither the physical strain nor the steroid treatment affect the cell morphology. The insensitivity of the A549 cell morphology to the physical strain and steroid treatment has been discussed in previous literature. For example, Liu et al. showed that the cell membranes, nuclei, lamellar bodies, and other fine structures were maintained under periodic mechanical stretch [9]. In addition, Rucka et al. reported that the morphological heterogeneity of A549 cells is not sensitive to dexamethasone treatment [25].

In order to quantitatively study the cells under the steroid treatment and under the designated physical stimulation mimicking lung physical microenvironment with specific strain range (from 2–9%), the cells on central chamber (x = 125–375 μm) are selected for SPC expression characterization by analyzing the fluorescence images as shown in Figure 5a. Figure 5b,c shows the normalized average intensity for A549 cells cultured in the microfluidic devices under the 4 different conditions after 12 h and 24 h, respectively. In Figure 5b, the normalized average intensities of A549 cells in Device B, C, and D are 8%, 6%, and 18% higher than that in Device A after 12-h experiment, respectively. The ANOVA analysis results indicate that no statistically significant differences between the results obtained from Device A, B, and C, although the normalized intensities are slightly higher for the cells cultured in Device B and C comparing to those cultured in Device A. In contrast, the intensities of the cells in Device D are statistically higher than those in Device A, B, and C. The results suggest that the treatment of dexamethasone or the physical stimulation on A549 cells for 12 h promotes the expression of SPC; however, the effects are limited comparing to the control experiments. In addition, the steroid treatment and the physical stimulation have similar effects on SPC expression of the A549 cells. In comparison, the SPC expression is significantly higher for the cells under the combination of the steroid treatment and physical stimulation indicating the synergic effects of both treatments on SPC expression of the A549 cells for 12-h experiments.

To further demonstrate the SPC expression of the A549 cells for longer treatments, Figure 5c shows the results of the 24-h cell experiments. The results indicate that the normalized average intensities of the A549 cells in Device B, C, and D are 10%, 27%, and 37% higher than that in Device A after the 24-h experiments, respectively. The results of the ANOVA analysis reveal there are significant differences among the results obtained from all four sets of experiments (Device A, B, C, and D). In the 24-h experiments, the SPC expression levels of the cells in the experimental conditions are all higher than those obtained in the control experiments. In the single treatment (either steroid treatment of physical stimulation) experiments, the cells under physical stimulation (Device C) have higher normalized average fluorescence intensities, indicating higher SPC expression level, comparing to those treated with the steroid (Device B). The results suggest that for 24-h treatment, the physical stimulation plays a more important role in promoting SPC expression of the A549 cells. Furthermore, the normalized average fluorescence intensities of the cells under the combined treatments (Device D with both steroid treatment and physical stimulation) are even higher than other conditions indicating even higher SPC expression level of the cells comparing to the cells under single treatments. The results again demonstrate the synergic effects of both treatments on SPC expression of the A549 cells. The similar experimental results have been mentioned in the previous studies. For example, Nakamura et al. reported that the increased SPC mRNA expression under the intermittent mechanical strain (5% elongation for 24-h) with dexamethasone treatment is higher than that without dexamethasone [10].

The experimental characterization results have confirmed that the developed device is capable of generating multiple well-controlled physical stimulations, including: cyclic strain and hydrostatic pressure with neglectable shear stress, which mimic in vivo physical microenvironments in lungs for in vitro cell culture. Furthermore, the cell experiment observation confirms the importance of the biomimetic microfluidic cell culture devices capable of reconstituting key physical microenvironments for in vitro cell studies. Further studies of cellular responses under more different physical stimulation combinations using the developed device as demonstrated in this paper can greatly help biologists elucidate influences of breathing movement under different normal or disease states. In addition, the experiments combined with drugs and other soluble factors can also be conducted using the developed device to investigate the effects of interactions between chemical and physical microenvironments on cellular behaviors. These characteristics warrant the reported biomimetic device a favorite candidate to conduct in vitro biomedical research on extensive pulmonary cells.

## 4. Conclusions

This paper reports a simple, but powerful, biomimetic microfluidic device capable of generating hydrostatic pressure and cyclic strain to provide a platform mimicking the lung physiological physical microenvironment for in vitro cell studies. The device is capable of providing an in vitro model with more in vivo-like microenvironments compared to conventional cell culture methods, and the device can be exploited for various pulmonary studies. For demonstration, in the experiments, the hydrostatic pressure applied to pneumocyte cells is used to mimic the microenvironment in fetal trachea during apneic periods on the saccular stag, and the physical surface strain applied upon a flexible PDMS membrane to stretch cells within the luminal cell culture chamber is used for simulating fetal breathing movement. The results demonstrate that the developed device can successful construct physical microenvironments similar to the physiological ones, and the cell experimental results suggest that physical stimulation may play a more important role in regulating the SPC expression level than the steroid treatment. The developed device can pave the way to better understand the cellular behaviors under various lung physiological conditions, and is promising for further translational studies.
